# Impact of COVID-19 lockdown in a biomedical research campus: A gender perspective analysis

**DOI:** 10.3389/fpsyg.2022.906072

**Published:** 2022-10-28

**Authors:** Nuria Izquierdo-Useros, Miguel Angel Marin Lopez, Marta Monguió-Tortajada, Jose A. Muñoz-Moreno, Cristina Agusti Benito, Sara Morón-López, Harvey Evans, Melisa Gualdrón-López, Jörg Müller, Julia G Prado

**Affiliations:** ^1^IrsiCaixa AIDS Research Institute, Badalona, Spain; ^2^Germans Trias i Pujol Research Institute (IGTP), Badalona, Spain; ^3^Cardiology Service, Germans Trias i Pujol University Hospital, Badalona, Spain; ^4^Fundació Lluita Contra la SIDA i les Malalties Infeccioses (FLS)-Infectious Diseases Service, Germans Trias i Pujol University Hospital, Badalona, Spain; ^5^Faculty of Psychology and Education Sciences, Universitat Oberta de Catalunya (UOC), Barcelona, Spain; ^6^Center “Estudis Epidemiològics Sobre les Infeccions de Transmissió Sexual i Sida de Catalunya” (CEEISCAT), Departament de Salut, Generalitat de Catalunya, Badalona, Spain; ^7^Spanish Consortium for Research on Epidemiology and Public Health (CIBERESP), Instituto de Salud Carlos III, Madrid, Spain; ^8^CIBER en Enfermedades Infecciosas, Madrid, Spain; ^9^ISGlobal, Hospital Clínic–Universitat de Barcelona, Barcelona, Spain; ^10^Internet Interdisciplinary Institute IN3, Universitat Oberta de Catalunya, Barcelona, Spain

**Keywords:** COVID-19, gender bias, scientific production, lockdown, dependents

## Abstract

From March to September 2020, researchers working at a biomedical scientific campus in Spain faced two lockdowns and various mobility restrictions that affected their social and professional lifestyles. The working group “Women in Science,” which acts as an independent observatory of scientific gender inequalities on campus launched an online survey to assess the impact of COVID-19 lockdowns on scientific activity, domestic and caregiving tasks, and psychological status. The survey revealed differences in scientific performance by gender: while male researchers participated in a larger number of scientific activities for career development, female researchers performed more invisible scientific tasks, including peer review or outreach activities. Mental impact was greater in researchers caring for children or dependents, and this was aggravated for women. Results spot a disproportionate impact of COVID-19 lockdowns on female scientific career development, and urges for equity measures to mitigate the consequences of an increase in the gender gap in biomedical sciences for current and future pandemics.

## Introduction

COVID-19 has changed our lives globally, and 2 years after this pandemic began, we are still trying to understand the real influence and impact that this prolonged experience is having on our lives. The continuous lockdowns and mobility restrictions imposed during these past months may have had different consequences, and gender equity could be one of the aspects profoundly affected. This may have particularly disturbed a broad scientific and clinical research community who has had to put their research on hold to quickly react to the COVID-19 pandemic to find new medical and scientific solutions.

The Women in Science (WiS) working group is a voluntary team of people employed by different research institutions devoted to healthcare and biomedical research located on the Can Ruti Campus.^1^ The biomedical research hub at the Can Ruti Campus centers its activity around the University Hospital Germans Trias i Pujol (HUGTiP) in Badalona, one of the biggest teaching hospitals in the Barcelona area (Spain), which provides state-of-the-art healthcare services to more than 2 M people. The Can Ruti Campus employs more than 900 people who devote their efforts to translational science. The WiS group works on Campus as an observatory and independent consulting agent while organizing activities to raise awareness of gender equity to promote the principles fostered by the Open Science initiative.^2^

The Spanish government issued a decree of a state of emergency from the March 14, 2020 until the September 14, 2020. During that period, the different research institutions based in the Can Ruti Campus faced two country-wide COVID-19 lockdowns and several mandatory restrictions that limited non-essential mobility and the number of researchers able to work in their laboratories and facilities. This emergency forced Can Ruti Campus scientists to work from home while facing new challenges in their life-work balance and continuous stress, considering that schools and high schools remained closed. During the period of lockdowns, the WIS group organized an internal survey to monitor the consequences that mobility restrictions could have had on career development, mental health, and wellbeing of the researchers working on Campus, particularly focusing on the backlash it could have on gender gap.

## Conceptual framework and hypotheses

The unequal distribution of domestic work and informal care responsibilities for children or dependent adults constitutes one of the contemporary societies’ central dimensions of gender inequality. According to recent statistics, across the EU, women bear the brunt of informal care work, including care for older people, people with disabilities, or children (Eurofound, 2017; European Institute for Gender Equality, 2020). In 2016, 13% of all working women compared to 9% of all working men in the EU provided informal care to older people or people with disabilities at least several times a week (Eurofound, 2017; European Institute for Gender Equality, 2020). A similar finding holds for informal care responsibilities for own children or grandchildren, with working women being more likely to be involved than working men across 24 EU Member States (Eurofound, 2017; European Institute for Gender Equality, 2020). Although time dedicated to informal care is converging between genders, “women continue to spend significantly more time than men on unpaid caring activities across the Global North and South, with estimates typically ranging from two to four times greater time investment” for women (Lightman and Kevins, 2021). As a result of the higher care responsibilities, women in employment carry the “double burden” of needing to perform on the job while also having to satisfy demanding parenting ideals. Work-life conflict and tensions are the result, as women are both expected to adhere to “ideal worker norms as though they do not have children,” while also being under pressure from “intensive mothering norms” which “expect women to parent as if they do not have careers” (Ward and Wolf-Wendel, 2016).

While the gendered nature of the work-life conflict (or balance) can be observed across the labor market in general, it is exacerbated in an academic context. This is because performance demands are “blurry,” following the rule “the more, the better,” at the same time that working conditions have become more precarious through the continued marketisation of academic and research activities (Bozzon et al., 2017; Nielsen, 2017; Huang et al., 2020). The academic career model requires to complete a series of sequential stages within a specified timeframe, building up a “rush hour” in terms of professional demands to obtain a doctorate, carry out research abroad, secure a post-doctoral position, and engage in intense competition for a tenure track position (European Science Foundation, 2009). Securing an independent position requires constant availability and visibility, i.e., a total commitment to the job through long working hours whose outcome or benefits are far from secure. Difficulties in adhering to this “ideal worker norm” produces work-life conflict resulting in a lower job-or career satisfaction, higher turnover intentions, or other stress-related outcomes (Sirgy and Lee, 2018) leading ultimately many women to abandon their science careers. There is robust evidence that ongoing care responsibilities tend to have a strong, negative impact on women’s careers (Friedman, 2015; Ahmad, 2017). As such, work-life conflict is a central explanatory factor for the continued gender imbalance observed in the academic field, where women are under-represented on the top of the career ladder. Although the details vary according to scientific discipline and countries, overall, in the EU-27, women represent 48.1% of doctoral students, while only 26.2% make it to the highest academic (grade A) positions (European Commission, 2021).

Given this overall situation of gender inequality in academic careers due to work-life balance issues, the question arises of which impact the COVID-19 lockdown has had in this respect. With schools and other (child) care services being closed down and support networks being disrupted, it stands to reason that women as primary cares would be disproportionately affected by the need to contain not only the additional educational and care tasks but also increased household work. Cross-country research, including the United States, Brazil, Denmark, Spain, and Canada, demonstrates a uniform picture of women and mothers, spending more time on childcare and household chores (Giurge et al., 2021). Although, men working from home increased their share of household-and care work in some cases, this has not ameliorated existing gender disparities. As Dunatchik et al. report (Dunatchik et al., 2021) from their nationally representative sample in the United States, 79% of mothers among all partnered couples indicated to be the primary responsible for housework (79%) and childcare (66%) compared to 28 and 24% of all fathers, respectively.

In the context of the present study, the gendered impact of the pandemic on academics will be examined, especially in relation to its potential effects on (Eurofound, 2017) scientific productivity, (European Institute for Gender Equality, 2020) other academic service work, and (European Institute for Gender Equality, 2020) mental health-related outcomes such as stress or burnout. These three aspects are especially pertinent for spelling out how work-life conflict during the pandemic can further aggravate gender inequalities in the academic workforce.

First, scientific publications remain the fundamental yard stick for advancing academic careers. At the same time, the reign of “publish-or-perish” has clear gendered undertones: women publish less than men on average. Despite discipline-and country-specific differences, men publish 13.2 papers during their entire career while women publish only 9.2 papers, i.e., 27% less (Huang et al., 2020). Although the possible factors explaining this “productivity puzzle” in science are pretty diverse—including, for example, insufficient disciplinary specialization (Leahey, 2006) or workplace climate (Settles et al., 2006), among others—work-life balance constitutes a central explanatory factor of the gender gap in scientific productivity (Eagly, 2020). The validity of this observation was confirmed as research on the productivity slump by women academics was published during the first month of the pandemic (King and Frederickson, 2020; Viglione, 2020; Squazzoni et al., 2021). As daycare centers and schools were closed, the available time dedicated to work—including writing papers—was significantly reduced for women with care responsibilities—adversely affecting career progression. Thus, we hypothesized that COVID-19 lockdowns could most profoundly impact the career development of female researchers and aimed to measure this potential impact in our research community.

Second, the distribution of academic workload is itself gendered. Research has consistently shown that women faculty spends more time on teaching-related activities, student advising or other service tasks while men spend more time on research (O’Meara et al., 2017). According to gendered role expectations in Western societies, men are usually seen as more agentic and competent than women, who are perceived as more communal-oriented and less competent (Fiske et al., 2002; Glick et al., 2004). These social stereotypes prescribe women as caretakers and men as leaders, thus unbalancing the distribution of academic workload. Women tend to take on precisely those tasks which are congruent with their gender roles, including other directed services such as work on committees (Porter, 2007), community services (Antonio et al., 2000), or teaching responsibilities (Link et al., 2008). We stipulate that these other-directed, service-oriented responsibilities during the pandemic will be maintained. Hence, we hypothesize that these other service tasks with lower beneficial impact on career development would still be associated with female researchers during COVID-19 lockdowns.

The third aspect of how the pandemic has had detrimental effects on gender equality in academia relates to the intensified work demand and its stress-related outcomes. Although a large part of society was shut down due to social distancing rules, work demands were intensified in the academic sector due to the need to adapt research processes and educational activities to epidemic restrictions and an online/remote work environment. While research on pre-pandemic work-life conflict has broadly shown that work-life conflict causes stress and burnout in general (Allen et al., 2000; Sirgy and Lee, 2018), these negative psychological outcomes can be expected to worsen during the pandemic. Indeed, during the pandemic, women were more at risk of depression than men (Nivakoski and Mascherini, 2021). Thirty percent of women and 34% of women with children report being mildly stressed compared to 19% of men—with and without kids (Zamarro and Prados, 2021). Given that academia has been singled out as a high-stress occupation, mental health issues will likely worsen for academic women and mothers due to the pandemic (Kinman and Jones, 2008; Morrish, 2019). Indeed, as the report by the Committee on Investigating the Potential Impacts of COVID-19 on the Careers of Women in Academic Science, Engineering, and Medicine, Committee on Women in Science, Engineering, and Medicine, Policy and Global Affairs, National Academies of Sciences, Engineering, and Medicine (2021) summarizes, the “[…] COVID-19 pandemic has exacerbated many stresses women in academia face under usual conditions” in terms of work-life balance and scientific productivity. The “pandemic burnout” is rampant in academia, with women again being hit hardest (Gewin, 2021). Hence, the final hypothesis we aimed to address with our survey was if female researchers on campus were more affected by stress than their male colleagues during COVID-19 lockdowns.

## Results

This survey took place from July to October 2020 and had 152 responders. Of note, the number of answers in the survey was similar to the number of people that engage in the annual activity organized by the WIS to commemorate the International Day of Women and Girls in Science. Three participants were excluded by invalid answers, leaving 149 responders, of whom two non-binary researchers were not analyzed, given the reduced sample size. Thus, 147 out of the 700 researchers on campus answered the survey (answer rate: 21.7%). Most responders were women (80%), concurring with the larger female presence on campus. The median age was 39.6 [IQR 30.5–45.9], half of the responders had children or someone in their care (55%), the education level was very high (72%), independently of gender, and the vast majority were originally from Spain (91%), with diverse sexualities (Table 1).

**Table 1:** Demographic characteristics of the scientists that respond to the survey.

	***n* (147)**	**Women (118)**	**Men (29)**	**p** ^**1**^
Age, median [IQR]	39,6 [30,5–45,9]	38,9 [20,5–44,0]	41,2 [31,7–47,8]	0,2073
**Family responsibilities**				
With people in charge, n (%)	81 (55,1)	64 (54,2)	17 (56,6)	0,7,038
Not in Charge, *n* (%)	26 (44,2)	53 (44,9)	12 (41,4)	
NA, *n* (%)	1 (0,7)	1 (0,8)	0	
**Professional research**^**2**^				
Yes, *n* (%)	59 (38,1)	45 (38,1)	14 (48,3)	0,3,182
No, *n* (%)	88 (59,9)	73 (61,9)	15 (51,7)	
**Country**				
Spain, *n* (%)	134 (91,2)	106 (89,8)	28 (96,6)	0,2,534
Other, *n* (%)	13 (8,8)	12 (10,2)	1 (3,4)	
**Income**				
501–1,000€, *n* (%)	2 (1,4)	2 (1,7)	0	0,4,765
1,001–1,500€, *n* (%)	44 (29,9)	37 (31,4)	7 (24,1)	
1,501–2000€, *n* (%)	47 (32,0)	38 (32,2)	9 (31,0)	
2001–4,000€, *n* (%)	43 (29,3)	31 (26,3)	12 (41,4)	
NA, *n* (%)	11 (7,5)	10 (8,5)	1 (3,4)	
**Employment**				
Full time, *n* (%)	137 (93,2)	108 (91,5)	29 (100)	0,2069
Part time, *n* (%)	6 (4,1)	6 (5,1)	0	
Other, *n* (%)	4 (2,7)	4 (3,4)	0	
**Education level**^**3**^				
Very High, *n* (%)	106 (72,1)	87 (73,2)	19 (65,5)	0,5,629
High, *n* (%)	28 (19,0)	20 (16,9)	8 (27,6)	
Medium, *n* (%)	8 (5,4)	6 (5,1)	2 (6,9)	
Low, *n* (%)	1 (0,7)	1 (0,8)	0	
Others, *n* (%)	4 (2,7)	4 (3,4)	0	
**Sexuality**				
Heterosexual, *n* (%)	128 (87,1)	104 (88,1)	23 (82,8)	
Homosexual, *n* (%)	7 (4,8)	2 (1,7)	5 (17,2)	0,0042
Bisexual, *n* (%)	6 (4,1)	6 (5,1)	0	
Other, *n* (%)	2 (1,4)	2 (1,7)	0	
NS/NC, *n* (%)	4 (2,7)	4 (3,4)	0	

1 Statistical differences according to Chi-squared test.

**Figure 1:**
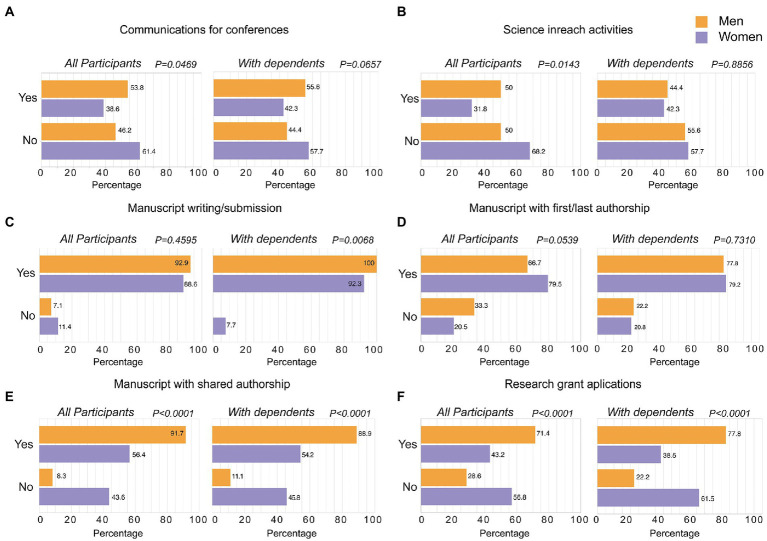
Impact of COVID-19 lockdowns in career development activities for scientists. **(A–F)** The indicated activities were analyzed in the subset of participants performing professional research activities (*n* = 59; left) and only those with professional research activities and children or dependents at care (*n* = 35; right). Numbers in each bar show the percentage of men (orange bars) and women (purple bars) who did (Yes) or not (No) each of the activities analyzed. Statistical differences in the distributions between men and women were analyzed with Fisher’s exact test.

**Figure 2:**
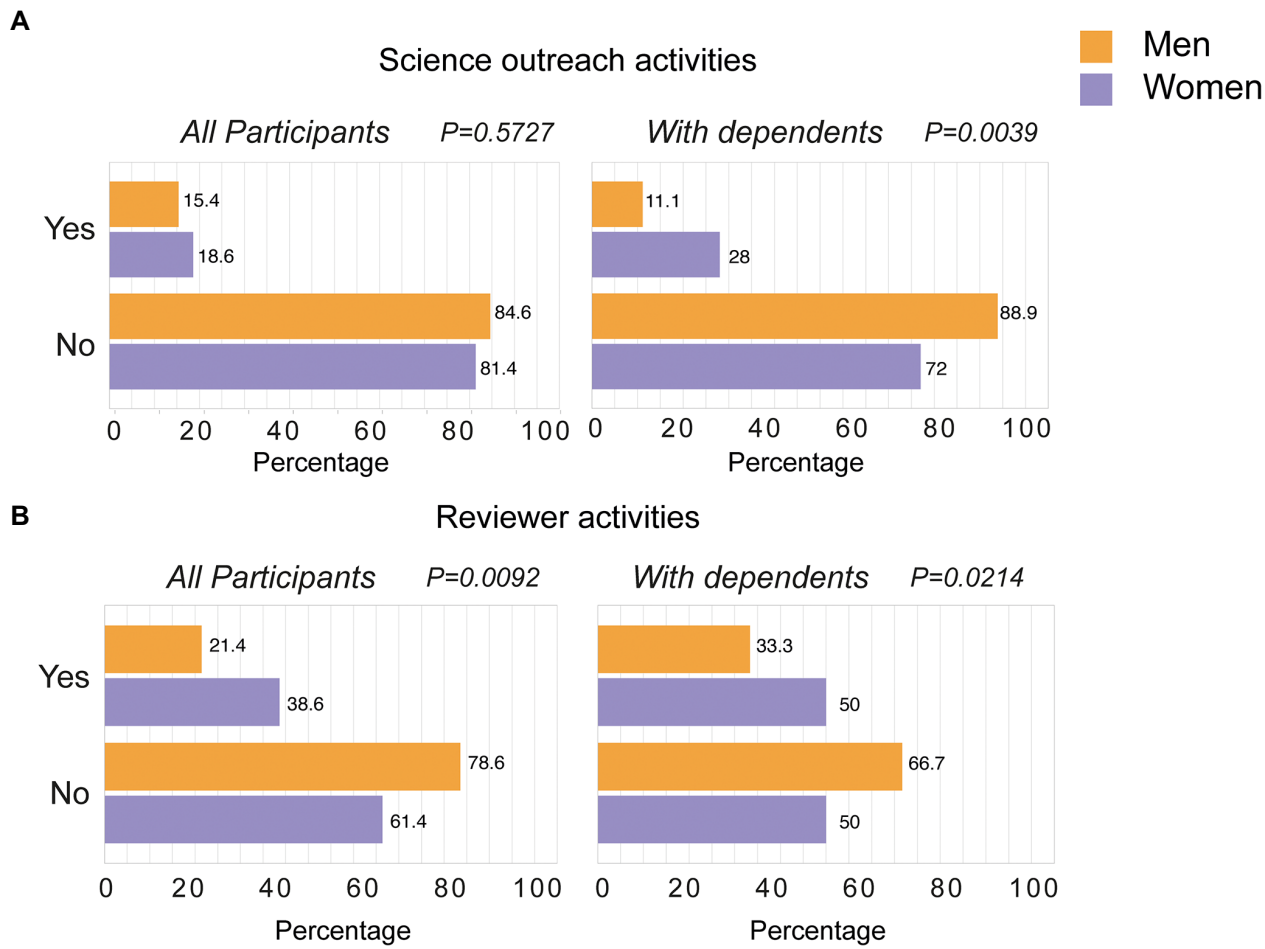
Impact of COVID-19 lockdowns in the participation on service work-oriented scientific tasks. **(A,B)** The indicated activities were analyzed in the subset of participants performing professional research activities (*n* = 59; left) and only those with professional research activities and children or dependents at care (*n* = 35; right). Numbers in each bar show the percentage of men (orange bars), and women (purple bars) who did (Yes) or not (No) in each of the activities analyzed. Statistical differences in the distributions between men and women were analyzed with Fisher’s exact test.

2 Answered performing active research activities during the study period, related to Figures 1, 2.

3 Education level based on International Standard Classification of Education (ISCED) categorization: Very high = ISCED level 7–8 (Doctoral, Master’s or equivalent); High = ISCED level 6 (Bachelor degree or equivalent); Medium = ISCED level 5 (Short-cycle tertiary education); and Low = ISCE level 1–4 (from Primary to Post-secondary non-tertiary education).

The first set of questions of the survey analyzed the impact of COVID-19 restrictions on scientific production, which is critical for career development. For this purpose, we focused on those participants reporting active professional research activities. The number of abstract submissions for conferences was higher in men than in women, although this statistical difference disappeared when we focused only on those researchers with children or dependents (Figure 1A). Dissemination of scientific results was mostly performed by male scientist, but again this difference was not significant when we focused on scientists with children or dependents (Figure 1B). Concerning manuscript submission, we found no overall gender differences (Figure 1C). However, this changed when we analyzed researchers with children/dependents, where male researchers were more productive than female colleagues (Figure 1C). When considering the first or last authorship in publications, we found no significant differences between genders (Figure 1D), what contrasts with prior reports covering more extended analysis periods (King and Frederickson, 2020). Focusing on the number of shared authorships, which reflects the networking ecosystem where scientists develop their careers, we found that male researchers appeared always more often as first or last authors and shared more authorships regardless of whether they had children/dependents or not (Figure 1E). Research grant applications and proposals were also presented more frequently by male researchers, and this was statistically significant for all scientists, including those with children and dependents (Figure 1F). Of note, differences found in scientific production within the groups of men and women were not increased when they had dependents in their charge. Gender differences were even lost for some aspects such as in-reach activities or manuscripts with first or last authorship when we focused on researchers with dependents. This could be explained because researchers with dependents were significantly older compared with the rest (Supplementary Figure 1), and hence a longer career trajectory may have compensated for the reduction on effective research working time associated to taking care of dependents during the COVID-19 lockdowns.

The next set of questions focused on activities that are commonly performed by scientists in an altruistic or service work-oriented manner, as experimental studies have found that women are more likely to be altruist than men (Capraro, 2015; Capraro and Marcelletti, 2015; Brañas-Garza et al., 2018), and these activities do not have a direct impact on career evaluation (Figure 2). Outreach scientific activities for educational purposes were performed more frequently by female scientists when they had children/dependents (Figure 2A). The reviewing activities for journals and grant panels were also performed by women more significantly than their male counterparts, regardless of whether or not they had children or dependents (Figure 2B). Thus, during the semester where two COVID-19 lockdowns took place and several mobility restrictions limited work on campus, the research activity of male researchers leading to recognition and career development (such as grant, paper or abstract submission, and recognition of scientific achievements *via* in-reach activities or number of papers with shared authorship) was greater than their female colleagues, who performed more altruistic activities, such as participating in outreach educational programs or peer-review activities. For service work-oriented activities, these gender differences were sometimes even more significant when we focused on those researchers with children or dependents, as happened for outreach activities.

**Figure 3:**
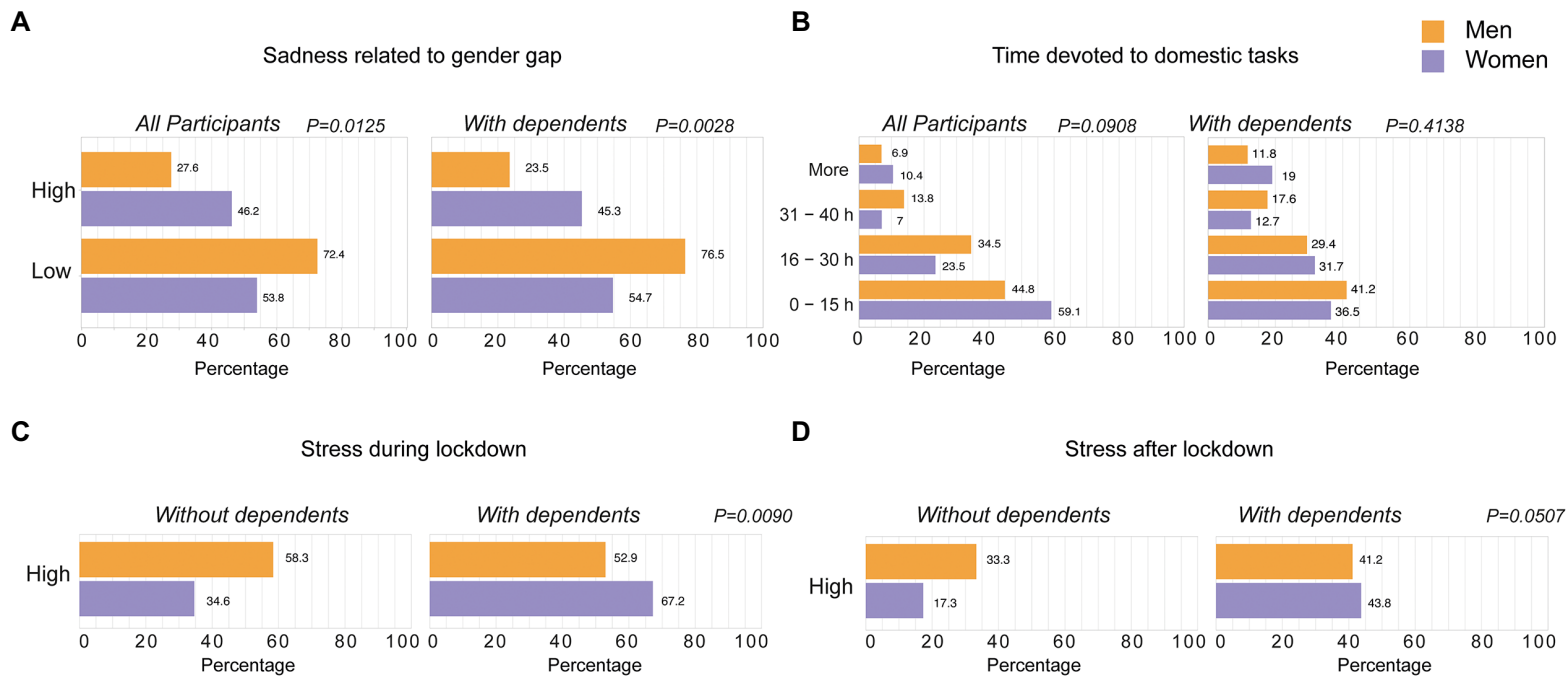
Impact of COVID-19 lockdowns on mental health and wellbeing of scientists. The self-reported mental health and wellbeing status or the indicated activities were analyzed **(A)** for all the participants (*n* = 146) or for those with children or dependents in care (*n* = 81). Numbers in each bar show the percentage of men (orange bars) and women (purple bars) who answered for each of the issues analyzed. The indicated wellbeing status was analyzed for those participants reporting high stress **(B)** during (*n* = 77) or **(C)** after lockdown (*n* = 48). Statistical differences in the distributions between men and women were analyzed with Fisher’s exact test **(A,C,D)** and Chi-squared test **(B)**.

Finally, we addressed the impact on mental health and wellbeing, and we only found statistical differences between male and female researchers when we analyzed sadness related to the expected gender gap challenges triggered by the pandemic, which was higher in women, and more significant in those having children or dependents (Figure 3A). Differences observed could not be attributed to the self-reported work time devoted to domestic and family care tasks, as we did not find significant differences between male and female researchers with children and dependents (Figure 3B). We did not detect statistical differences between male and female researchers when we analyzed variables such as perceived stress during or after lockdown or anxiety for future gender inequities as a consequence of the pandemic (data not shown). However, for those individuals with reported stress due to domestic and family care activities, female researchers with children or dependents had significantly more stress than female researchers lacking these responsibilities both during and after lockdown (Figures 3C,D). Thus, having children or people in their charge had a greater impact on women, who had more elevated levels of perceived stress due to domestic and family care during and after lockdown.

## Discussion

This study offers a valuable snapshot of the effects of the COVID-19 pandemic on the research outcomes and psychological impact on scientists in Spain. The survey performed during the worst semester of the pandemic provides an overview of the toll taken on all the researchers working in our campus who had to deal with striving to continue their careers and projects. The principal limitation of the study is the opportunistic sampling design of the survey focus on a particular research environment, making the results difficult to generalize to other research institutions in our region or in other areas of Spain. Yet, the results obtained herein align with those reported in distant geographical areas such as the United States (Myers et al., 2020; Subramanya et al., 2020; Sloane and Zimmerman, 2021). Another limitation is the low return rate for questionnaires (21.7%), but this response rate is, however, similar to that seen in other surveys of analogous study populations (Alonso-Flores and Moreno-Castro, 2018; Alonso et al., 2021). We also acknowledge the delay in publishing the results from this survey, which had to be postponed due to the pandemic duties of the WIS members. Yet, this analysis provides key insights into pandemic toll, and will be central to monitor the status and foster the wellbeing of our scientific community in the near future, where we will be affected by the challenges of future pandemics.

The study also underscores that not all researchers were equally affected, as female scientists performed less career development activities than their male counterparts, and this trend was observed regardless of the presence of family dependents. Our findings align with prior studies on gender gap in scientific output that linked detrimental differences observed for women on credited attribution rather than to the real scientific contribution of researchers (Ross et al., 2022). Previous reports have also shown greater psychological and emotional distress in women, mainly when they are young and involved in healthcare activities (Rodríguez-Fernández et al., 2021). Thus, our results add to the growing body of evidence suggesting a psychological and emotional vulnerability for women as a result of the pandemic (Almeida et al., 2020; Farrés et al., 2021). The differences found here may be even greater than the ones actually reported, as people who participated in the survey are most likely aligned with the goals pursued by the WIS and support gender equity on campus, reflecting researchers that have awareness of gender bias and are most likely to act accordingly. This may explain why we found that both female and male researchers devoted a similar amount of time to family/dependent care and household maintenance. Yet, the mental impact was affecting more female researchers with family responsibilities.

Overall, female researchers have experienced a decreased participation in career development activities, along with an increased participation in those tasks perceived as altruistic combined with greater stress associated to family care. These three significant differences clearly identify the main gender gap problems encountered in a research campus. Disparities may be attributed to the introjection of gender stereotypes by female researchers, who were more likely to devote their time to altruistic duties not directly linked to career promotion in combination to the burnout experienced during care for dependents. Our results align to other studies performed in Spain, where being a woman was found to be a risk factor for higher stress levels during the pandemic (Alonso et al., 2021; Farrés et al., 2021; Rodríguez-Domínguez et al., 2022). Spain is ranking within the top 20 countries with better global gender-gap index at the recently released report by the World Economic Forum, and can be considered as one of the most equal economies in 2022. Yet, lower-income salaries associated to female workers and imbalance between family and work life was a stronger predictor of stress during COVID-19 lockdowns in Spain, even stronger than having children or dependents (Rodríguez-Domínguez et al., 2022). Although similar findings have been found by international committees assessing the impact of COVID-19 on Women in the STEM in the USA (Committee on Investigating the Potential Impacts of COVID-19 on the Careers of Women in Academic Science, Engineering, and Medicine, Committee on Women in Science, Engineering, and Medicine, Policy and Global Affairs, National Academies of Sciences, Engineering, and Medicine, 2021) or in Asia and the Pacific (Australian Academy of Science, 2021), future work will need to address the impact of local culture in the differences found in our study.

Differences found herein represent opportunities for institutional leaders, policy makers, funders, and governments to offer counter measures to accelerate changes to reduce gender inequity. The WiS observatory group proposes the implementation of mentoring activities to promote and raise awareness in female researchers of the need to actively participate in activities linked to career development along with changes in current evaluation guidelines to include outreach activities and article peer-reviewing as parameters for funding grants and assigning contracts (Davies et al., 2021). Also, it will be a key to extend the period of funded activities for those researchers who were not able to work during lockdowns, and take these incidences into account for future grant applications. We will continue to monitor the consequences on career development for female scientists to adapt and respond to future challenges while disseminating our findings. In particular, we hope that implementing these measures on campus could contribute to mitigate burnout rates (Matulevicius et al., 2021). Although it is likely that the pandemic has aggravated the differences observed, as previously reported by other global studies (Flor et al., 2022), this cannot be concluded from the current survey. As gender gap differences are at the core of our scientific community and reflect a historical *status quo* that we have not yet been able to overcome, we have now an opportunity to reflect and implement solutions for the problems identified here.

## Methodology

The study protocol was approved by the Ethics Committee of the HUGTIP PI-21-214. The survey was online, self-administrated, and anonymous. The confidentiality of the subjects included in the study was guaranteed in accordance with the provisions of the current regulations on data protection law [Regulation (EU) 2016/679 of the European Parliament and of the Council of April 27, 2016; and the Spanish Organic Law 3/2018, of December 5].

The survey was advertised by mailing lists that covered five scientific research Institutes on the campus (IGTP, IrsiCaixa, Hospital Germans Trias i Pujol, FLS,and CEEISCAT), *via* equity/workers committees and communication departments of each research institute, on the institutional webpage of the Germans Trias i Pujol Health Science Research Institute (IGTP), and *via* personal interactions of the members of WIS.

The full questionnaire is presented as Supplementary material and was developed by a transdisciplinary team formed by basic, clinical, and public health researchers, men and women, and members of the WiS. Topic areas and items covered by the survey were evaluated by the WiS, focusing on interest, acceptability by potential responders, and ranked priority. The questionnaire was organized into four conceptual areas. The first area included sociodemographic information, such as age, city of residence, country of birth and year of arrival to Spain, gender, sexual orientation, cohabitants, level of education, employment situation, professional category, monthly income, and number of dependents (children and others). The answers related to these demographic characteristics are summarized in Table 1 and were used to calculate statistical differences using a Chi-squared test.

The second area was related to working hours, household duties, and free-time activities and included aspects such as time dedicated to care for others/household tasks, available support to execute these tasks, time working outside the home, time teleworking, and time for leisure. We also accounted for scientific production and activities in another area of the questionnaire, focusing on those with a direct positive impact on career development, such as publications or research grants, but also on those that are time-consuming and do not directly benefit professional growth. We took into account the number of written communications for conferences, manuscripts as first/last/or corresponding author, reviewer activities, dissemination activities, grants submitted as principal investigator, or number of projects awarded. Answers to all these sections were treated categorically, considering binomial answers (Yes/No). This categorization was used to calculate the percentage for the comparison of these groups using the indicated tests for each panel.

The final section of our survey was designed to study the mental health of the responders and elaborated *ad hoc* by the WiS group. Possible answers were however comparable to those included in other questionnaires widely used in the field of mental health (e.g., GAD-7, for anxiety disorders; or PHQ-4, for depression and anxiety). A total of 10 items assessing the concern, sadness, perceived stress, and anxiety resulting from the pandemic lockdowns were analyzed to address the mental health status. Specifically, these questions evaluated sadness related to the expected gender gap challenges triggered by the pandemic, perceived stress during or after the COVID-19 lockdown, or anxiety for future gender inequities as a consequence of the pandemic. Answers were treated categorically, considering low levels for the two initial choices (“not at all” and “mildly”) and high levels for the two final choices (“mostly” and “completely”). This categorization was used to calculate the percentage for the comparison of these groups using the indicated tests for each panel.

A descriptive analysis was carried out, comparing answers by men and women, taking into account whether they have children or not or someone in their care (dependents). Participants reporting active professional research activities were considered for the set of questions related to scientific production. This excluded nurses, lab technicians, project managers, and predoctoral students that did not actively participate in the professional research activities analyzed in this part of the survey. Statistical differences in frequency distribution data were calculated using the Chi-squared test or Fisher’s exact test and a significance level of 5%, using the GraphPad Prism v9.0.1 and SPSS software. Plots were generated using RStudio (v1.4) and the ggplot2 package (Wickham, 2009).

## Data availability statement

The original contributions presented in the study are included in the article/Supplementary material, further inquiries can be directed to the corresponding authors.

## Ethics statement

The studies involving human participants were reviewed and approved by Ethics Committee of the HUGTIP (PI-21-214). Written informed consent for participation was not required for this study in accordance with the national legislation and the institutional requirements.

## Author contributions

NI-U, ML, MM-T, JM-M, CB, SM-L, HE, MG-L, JM, and JP conceived the study, designed the questionnaire, and analyzed the data. ML, MM-T, and JM-M made the figures. JM elaborated the conceptual framework. NI-U and JP wrote the paper. All authors contributed to the article and approved the submitted version.

## Conflict of interest

The authors declare that the research was conducted in the absence of any commercial or financial relationships that could be construed as a potential conflict of interest.

## Publisher’s note

All claims expressed in this article are solely those of the authors and do not necessarily represent those of their affiliated organizations, or those of the publisher, the editors and the reviewers. Any product that may be evaluated in this article, or claim that may be made by its manufacturer, is not guaranteed or endorsed by the publisher.

## Footnotes

^1^ http://www.canruticampus.cat

^2^ https://ec.europa.eu/info/research-and-innovation/strategy/strategy-2020-2024/our-digital-future/open-science_en

## Supplementary material

SUPPLEMENTARY FIGURE S1 Violin plots showing the age difference between participants performing professional research activities without (*n* = 19 women; *n* = 7 men) or with children or dependents in care (*n* = 26 women; *n* = 7 men). Statistical differences assessed by two-way ANOVA with Tukey’s post-hoc test; ***p* < 0.01.
