# Supplementary figures and images for: Impact of COVID-19 lockdown in a biomedical research campus: A gender perspective analysis
Source: Front Psychol. 2022 Oct 28;13:906072. doi: 10.3389/fpsyg.2022.906072 (PMC9650053; doi:10.3389/fpsyg.2022.906072)

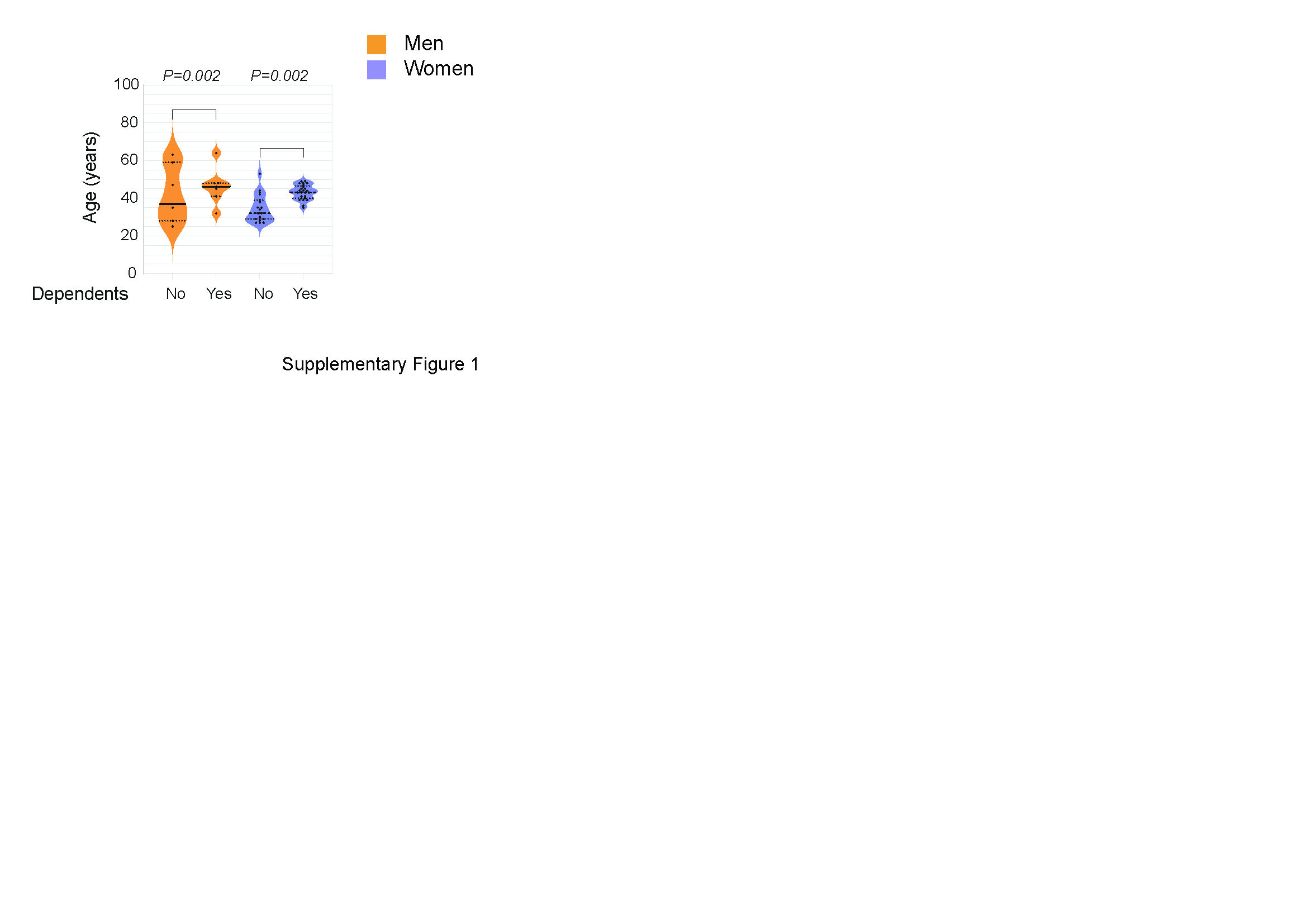

Supplement: SUPPLEMENTARY FIGURE S1 — Violin plots showing the age difference between participants performing professional research activities without (n = 19 women; n = 7 men) or with children or dependents in care (n = 26 women; n = 7 men). Statistical differences assessed by two-way ANOVA with Tukey’s post-hoc test; **p < 0.01. [file Image_1.tiff]
