# Supplementary material for: Impact of COVID-19 lockdown in a biomedical research campus: A gender perspective analysis
Source: Front Psychol. 2022 Oct 28;13:906072. doi: 10.3389/fpsyg.2022.906072 (PMC9650053; doi:10.3389/fpsyg.2022.906072)
Supplement: Supplementary file 2 [file Data_Sheet_2.docx]

Working group: Women in Science Can Ruti

Assessment of the Impact of the COVID-19 Pandemic Associated with Gender on Staff at Can Ruti Campus

This survey is aimed at staff working in a healthcare or research institution on the Can Ruti Campus. The survey is voluntary, anonymous and confidential. The Women in Science working group will report on the results once the survey has been analyzed.

Objectives:

1. To study the differential impact of care-associated work between men and women of the research staff of the Can Ruti Campus during the COVID-19 crisis.

2. To study the proportion of staff at the Can Ruti Campus during the COVID-19 crisis who have reduced their working hours or who plan to do so in the coming months.

3. To estimate the impact of COVID-19 on the scientific production (articles, communications to congresses, reports) of the research staff of the Can Ruti Campus.

4. Estimate the impact of COVID-19 on the participation in calls for projects of the research staff of the Can Ruti Campus.

5. Study the factors associated with the changes in scientific production and participation in calls of the research staff of the Can Ruti Campus.

6. Identify the concerns of the research staff of the Can Ruti Campus regarding the return to face-to-face and post-confinement work.

7. Know the impact of COVID19 on mood and stress in relation to possible gender inequalities.

Do you work at the Can Ruti Campus? Yes/No

1. Personal data
   1. Date of birth
   2. City and country of birth
   3. How long have you lived in Spain?
   4. Biological sex at birth.
      1. Woman
      2. Man
   5. Sex you identify with?
      1. Woman
      2. Man
      3. Transgender woman
      4. Transgender man
      5. No binary
      6. Other
      7. I don’t know/ I’d rather not answer
   6. How do you consider yourself?
      1. Heterosexual
      2. Homosexual
      3. Bisexual
      4. Others
      5. I don’t know/ I’d rather not answer
   7. Which is the situation that best defines who you live with?
      1. Alone
      2. With my partner
      3. With my family (parents and other adult relatives)
      4. Children
      5. Friends
      6. Other adults
      7. Others
      8. NA
   8. What is the highest level of education you have completed?
      1. Incomplete elementary
      2. Complete elementary
      3. High school
      4. Baccalaureate
      5. School certificate (FPI)
      6. College (major)
      7. University (degree)
      8. PhD/MIR
      9. Others
      10. I don’t know/ I’d rather not answer
2. Family and work data
   1. What is your employment situation?
      1. Full-time
      2. Part-time
      3. Self-employed
      4. Unemployed
      5. Student
      6. Retired
      7. Sick leave
      8. I don’t know/ I’d rather not answer
   2. What is your professional category?
      1. General services and maintenance
      2. Management and administration
      3. Care staff
         1. No
         2. Head/ Chief
         3. Physician
         4. Resident doctor
         5. Nurse
         6. Nursing assistant
      4. Support research staff
         1. No
         2. Laboratory technician
         3. Specialist
         4. Project manager
      5. Research staff
         1. No
         2. Director
         3. Head chief
         4. Principal investigator
         5. Research associate
      6. Research student
      7. Other
      8. I don’t know/ I’d rather not answer
   3. What is your net monthly income?
      1. No income
      2. Less than 500 €
      3. 501 – 1000 €
      4. 1001 – 1500 €
      5. 1501 – 2000 €
      6. 2001 – 4000 €
      7. More than 4000 €
      8. I don’t know/ I’d rather not answer
   4. How many people were dependent on your income (including yourself)?
      1. (1, 2, 3…)
   5. Does any of these individuals have special needs?
   6. Which ones?
   7. How many children do you have?
      1. 0
      2. 1
      3. 2
      4. 3
      5. More than 3
      6. I don’t know/ I’d rather not answer
   8. How old are your children?
   9. How many people older than you do you take care of?
      1. 0
      2. 1
      3. 2
      4. 3
      5. More than 3
      6. I don’t know/ I’d rather not answer
3. Situation during the confinement
   1. How many hours per week did you spend taking care of others or doing household tasks?
   2. How many hours per week did you have family help/support to take care of children and/or dependents or to do household tasks?
   3. How many hours per week did you have professional help/support to take care of children and/or dependents or to do household tasks?
   4. How many hours per week did you spend having fun (meeting with friends, video calls, doing sports, reading, watching TV, etc.)?
   5. How many hours per week did you work outside your home before the confinement ?
   6. Did you telework/work from home during the confinement ?
   7. How many hours per week did you telework?
   8. Did you have to do clinical work during the confinement ?
   9. How many hours per week did you have to do clinical work?
4. Research during the confinement
   1. Do you do research?
   2. How many times did you take part in writing communications for conferences?
   3. Did you participate in writing or submitting manuscripts (both pre-print and scientific journals)?
   4. How many manuscripts did you sign as first, last or corresponding author?
   5. From those, how many were co-authored?
   6. Did you participated as a reviewer of scientific publications?
5. Dissemination during the confinement
   1. How many times did you participate as a speaker or were you interviewed for scientific dissemination activities (webinars, radio, TV or newspapers interviews, etc.)?
   2. How many times did you participate as a speaker in scientific dissemination activities (conferences, seminars, webinars, workshops, etc.)?
6. Projects during the confinement
   1. How many times did you participate in thinking, writing and submitting proposals to grant applications?
   2. From those, in how many were you the principal investigator (PI)?
   3. From those, in how many were you the co-PI?
   4. Which is the status of the submitted projects?
      1. Awarded
      2. Denied
      3. Pending
7. Health during the confinement
   1. Are you or have you been infected with COVID-19?
      1. Yes, I did not present symptoms, but I had a positive test
      2. Yes, I had symptoms and I had a positive test
      3. No, I have not had symptoms
      4. No, I have not had symptoms and I had a negative test
      5. I don’t know/ I’d rather not answer
   2. If you have had COVID-19 symptoms, indicate which ones (you can select more than one answer):
      1. Fever
      2. Dry cough
      3. Difficulty to breath
      4. Sore throat
      5. Fatigue
      6. Headache
      7. Loss of smell
      8. Loss of taste
      9. Diarrhea
      10. Vomits
      11. Conjunctivitis
      12. I don’t know/ I’d rather not answer
   3. Other symptoms
   4. How have this symptoms limit your daily life?
      1. Nothing
      2. Something
      3. A lot
      4. Completely
   5. If you have had COVID-19 symptoms, were you isolated?
   6. How many days were you isolated?
   7. If you have had COVID-19 symptoms, did you have to be admitted to hospital?
   8. If you were admitted, did you have to be transferred to the ICU?
   9. Have you had a relative or close friend in a critical situation due to COVID-19 (admitted to ICU, risk group, etc.)?
8. Impact
   1. To what extent are you concerned that the COVID-19 crisis could negatively affect the development of your professional career due to an increase in the number of hours dedicated to family care work?
      1. Not at all
      2. Mildly
      3. Mostly
      4. Completely
   2. How often do you think about the negative impact this crisis can have on increasing gender inequalities?
      1. Not at all
      2. Mildly
      3. Mostly
      4. Completely
   3. Do you feel sad or discouraged as a result of the impact that the crisis may have on gender inequality in the specific area of your research?
      1. Not at all
      2. Mildly
      3. Mostly
      4. Completely
9. Stress
   1. How would you rate your stress level during confinement in relation to the research tasks you carry out?
      1. Not at all
      2. Mildly
      3. Mostly
      4. Completely
   2. How would you rate your stress level during confinement in relation to the associated care tasks that you have developed?
      1. Not at all
      2. Mildly
      3. Mostly
      4. Completely
   3. To what degree do you notice more anxiety or anguish about the possible increase in gender inequalities in your professional field after the COVID-19 crises?
      1. Not at all
      2. Mildly
      3. Mostly
      4. Completely
   4. How would you rate your stress level at the present time in relation to the research tasks that you have to develop?
      1. Not at all
      2. Mildly
      3. Mostly
      4. Completely
   5. How would you rate your stress level at the present time in relation to the care work that you have to develop?
      1. Not at all
      2. Mildly
      3. Mostly
      4. Completely
   6. How would you rate your stress level at the current moment in relation to the reconciliation between your care work and the research tasks that you have developed?
      1. Not at all
      2. Mildly
      3. Mostly
      4. Completely
   7. Are you considering asking for a reduction in working hours to care for your child / children or seniors in your care in the event that schools / day centers or similar or similar close again?
      1. Yes
      2. No

Thank you,

The Can Ruti Women in Science Working Group will disseminate the results once the survey has been analyzed.
